# Supplementary material for: Identification of Key Biomarkers Related to Lipid Metabolism in Acute Pancreatitis and Their Regulatory Mechanisms Based on Bioinformatics and Machine Learning
Source: Biomedicines. 2025 Aug 31;13(9):2132. doi: 10.3390/biomedicines13092132 (PMC12467098; doi:10.3390/biomedicines13092132)
Supplement: Supplementary file 1 [file biomedicines-13-02132-s001.zip › biomedicines-3779717-supplementary.pdf]

Supplementary materials

Table S1 Key criteria for selecting datasets

Species Consistency	Group Completeness	Data Quality Control	Phenotypic Relevance
Prioritized mouse-derived datasets to align with our subsequent AP mouse model validation system, thereby minimizing interspecies variability	Required clear "healthy control" and "acute pancreatitis (AP)" groups with matched sample sizes to avoid group imbalance-induced bias	Selected datasets with:Raw data availability；Explicit platform information (e.g., GPL339, GPL6246)；No evident technical batch effects	Focused on:Pancreatic tissue samples；AP-specific pathological processes (excluding studies solely addressing complications or other tissues)

Table S2 Dataset details

Dataset	Methods of inducing AP	Mouse strain	Gene background	Stringent criteria
GSE3644	All three datasets used the same method of AP induction by rain frogin injection, which may be adjusted according to the different injection time points to eliminate the interference of different AP induction methods on the screening results to the greatest extent.	C57BL/6	Mist KO	①Mouse pancreatic RNA expression profiles ②Balanced sample sizes (6-9 controls vs. 5-8 AP cases) ③Batch-effect correctable via sva package (post-preprocessing PCA showed good intra-group clustering, Fig. 2B)
GSE65146		C57BL/6	KrasG12D-mutated mice	
GSE121038		C57BL/6	BCT KO	

Table S3 Pathological Scoring Criteria

Pathological Status	0 Point(s)	1 Point(s)	2 Point(s)	3 Point(s)
Edema	No significant fluid accumulation; clear tissue architecture	Mild interstitial fluid accumulation with preserved tissue architecture	Severe interstitial fluid accumulation with significant architectural distortion	Mild interstitial fluid accumulation with preserved tissue architecture
Inflammatory Cell Infiltration	No infiltration	1~4 inflammatory foci per high-power field (HPF), predominantly lymphocytes and macrophages	5~10 inflammatory foci per HPF, involving lymphocytes, macrophages, and neutrophils.	>10 inflammatory foci per HPF, with extensive mixed inflammatory cell infiltration.
Necrosis	No necrosis; intact tissue architecture.	<30% tissue necrosis.	30%~50% tissue necrosis.	>50% tissue necrosis.

Table S4 Primer sequences for RT-qPCR of genes

Gene	Forward (5'-3')	Reverse (5'-3')
Amacr	GCCAATCGGGAATTTTCTCCA	GTAGGGGTTCTCACCGCTT
Cyp39a1	CACTGCATGAGCGACTGTAT	TTCAGTTAGCGGCCCAGTAAA
Echs1	AGCAAGTTCCTGAGCCACTG	ACCCCCACCAAGAGCATAAC
Gpd2	AGGGGACGCCGGATCTAA	ATATGCCAGGCTCACTTGCTT
Osbpl9	TATACCAGCCTAGTCCCTTGGA	CTGCTCTGACTTACACAACTGAG
Acsl4	TGTGGACAATAAGGCTATCA	TGGTCTACTTGGAGGAATG
Mcee	AGATCCGTAGTCTGAGTGATGAG	CACAGTCTTTGGGATGGAGGA

Table S5 Experimental drugs and experimental reagents

Reagent name	Item number	Source
Nembutal	WS20190429	Sinopharm chemical Reagent Co., Ltd.
Sodium Taurocholate	243338B	Adamas
4% Paraformaldehyde	BL539A	Biosharp
Hematoxylin-Eosin(HE) Stain Kit	G1120	Beijing Solarbio Science & Technology Co., Ltd.
Anhydrous ethanol	10009218	Sinopharm chemical Reagent Co., Ltd.
Xylene	10023418	Sinopharm chemical Reagent Co., Ltd.
Paraffin	39601095	Leica
Trizol	R401-01	Nanjing Vazyme Biotech Co., Ltd
Trichloromethane	10006818	Sinopharm chemical Reagent Co., Ltd.
Isopropanol	40064360	Sinopharm chemical Reagent Co., Ltd.
75% ethanol	80176998	Sinopharm chemical Reagent Co., Ltd.
DEPC Water	G8010	Adamas
RNase Free ddH2O	C8023	Adamas
HiScript III All-in-one RT SuperMix Perfect for qPCR	R333	Nanjing Vazyme Biotech Co., Ltd
ChamQ Universal SYBR qPCR Master Mix	Q711	Nanjing Vazyme Biotech Co., Ltd

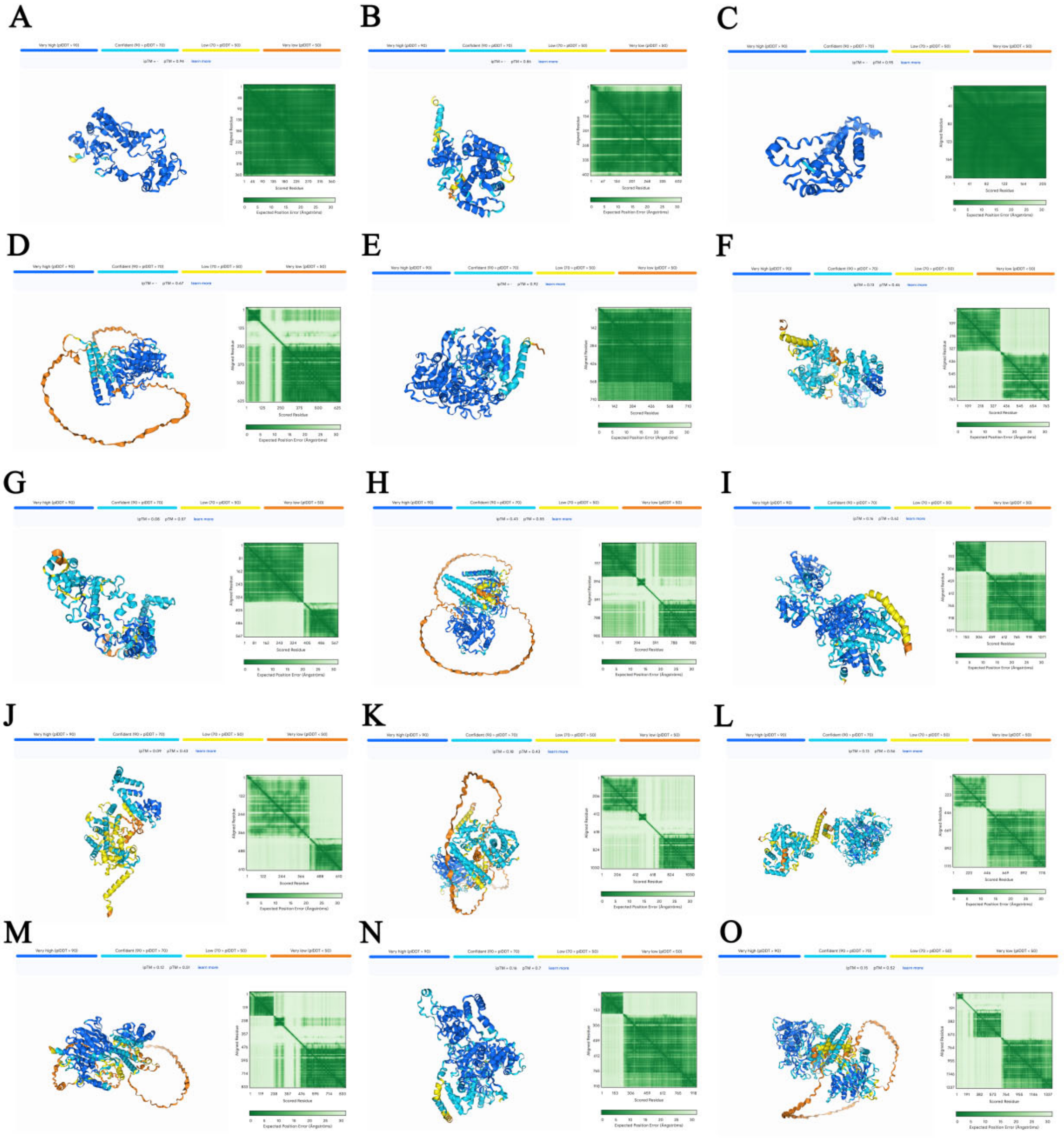

FigureS1 Protein structure prediction results (A-E) Amacr, Cyp39a1, Echs1, Osbpl9, Acsl4 protein structure prediction (F-O) Amacr&Cyp39a1, Amacr&Echs1, Amacr&Osbpl9, Amacr&Acsl4, Cyp39a1&Echs1, Cyp39a1&Osbpl9, Cyp39a1&Acsl4, Echs1&Osbpl9, Echs1&Acsl4、Osbpl9&Acsl4
